# Effects of Management Intervention on Post-Disturbance Community Composition: An Experimental Analysis Using Bayesian Hierarchical Models

**DOI:** 10.1371/journal.pone.0059900

**Published:** 2013-03-22

**Authors:** Jack Giovanini, Andrew J. Kroll, Jay E. Jones, Bob Altman, Edward B. Arnett

**Affiliations:** 1 Statistics, Mathematics, and Operations Research, Weyerhaeuser NR, Federal Way, Washington, United States of America; 2 Timberlands Technology, Weyerhaeuser NR, Federal Way, Washington, United States of America; 3 American Bird Conservancy, Corvallis, Oregon, United States of America; 4 Theodore Roosevelt Conservation Partnership, Washington, DC, United States of America; Duke University, United States of America

## Abstract

As human demand for ecosystem products increases, management intervention may become more frequent after environmental disturbances. Evaluations of ecological responses to cumulative effects of management interventions and natural disturbances provide critical decision-support tools for managers who strive to balance environmental conservation and economic development. We conducted an experiment to evaluate the effects of salvage logging on avian community composition in lodgepole pine (*Pinus contorta*) forests affected by beetle outbreaks in Oregon, USA, 1996–1998. Treatments consisted of the removal of lodgepole pine snags only, and live trees were not harvested. We used a Bayesian hierarchical model to quantify occupancy dynamics for 27 breeding species, while accounting for variation in the detection process. We examined how magnitude and precision of treatment effects varied when incorporating prior information from a separate intervention study that occurred in a similar ecological system. Regardless of which prior we evaluated, we found no evidence that the harvest treatment had a negative impact on species richness, with an estimated average of 0.2–2.2 more species in harvested stands than unharvested stands. Estimated average similarity between control and treatment stands ranged from 0.82–0.87 (1 indicating complete similarity between a pair of stands) and suggested that treatment stands did not contain novel assemblies of species responding to the harvesting prescription. Estimated treatment effects were positive for twenty-four (90%) of the species, although the credible intervals contained 0 in all cases. These results suggest that, unlike most post-fire salvage logging prescriptions, selective harvesting after beetle outbreaks may meet multiple management objectives, including the maintenance of avian community richness comparable to what is found in unharvested stands. Our results provide managers with prescription alternatives to respond to severe beetle outbreaks that continue to occur across extensive portions of the dry forests of western North America.

## Introduction

Large-scale environmental disturbances such as floods, fires, and insect outbreaks can influence species distributions, community composition, and ecosystem processes [1], [2]. Institutional responses to environmental disturbances seek to maintain populations and communities of native organisms, control exotic organisms, and reduce the risk of further disturbances and potential environmental degradation [3], [4], [5]. However, recovery of economic value in order to reduce detrimental societal impacts is often a primary consideration [6], [7], [8]. In so doing, management responses may exert additive or multiplicative effects on ecological processes beyond those caused by the disturbances themselves which, in turn, may have been influenced by previous anthropogenic activities [9], [10]. As human demand for ecosystem products increases, management interventions after disturbances may occur more frequently [11], [12]. In addition, increases in size and frequency of natural disturbances due to climate change are predicted [13], [14], [15]. Increased frequency of both natural and anthropogenic disturbances has unknown consequences for ecosystem dynamics. To prepare for these forecasted changes, more information is needed on the range of ecological responses to cumulative effects of anthropogenic and natural disturbances.

Forests disturbed by fire, extreme wind events, or insect infestations are often harvested to capture remaining economic value of standing and down timber. So called salvage-logging is a controversial conservation and management issue worldwide [10], [16]. Potential ecological effects of salvage logging include changes in vertebrate and invertebrate populations [17], [18]; stand regeneration rates [19], [20]; ecosystem processes [21], [22], [23]; and landscape pattern [24].

The broad variation observed in ecological responses to salvage logging suggests that interactions between ecological settings, disturbance agents, and harvesting methods are common. However, responses of individual species and communities of vertebrates to salvage logging generally reflect the structural complexity of an individual stand [16], [17], [25] which is itself a function of both the disturbance agent as well as the harvesting method that was used to conduct the salvage logging. For example, the number and condition of structures (e.g., shrub cover, number of snags, number of live trees) remaining when harvesting commences will be very different after fires [26], [27], wind storms [6], [28], and beetle outbreaks [24], [29]. Additionally, different harvest treatments, e.g., clearcutting vs. partial retention [30], [31], and systems, e.g., cable vs. ground logging [32], can yield different ecological responses [33], [34]. Finally, alternative harvest systems incur varying costs [30], [35], a primary concern given that salvage operations attempt to recoup economic value from disturbed stands.

We used a randomized-block experiment to study avian community responses to salvage logging of beetle-killed lodgepole pine (*Pinus contorta*) forests from 1996–1998 in Oregon, USA. We used recently developed statistical methods to estimate changes in species occupancy and community composition, rather than naïve estimators that are confounded with the detection process for individual species [36], [37]. Differences in vocalization type and rate, behavior, and habitat type can influence avian detection rates [38], [39]. Ignoring the detection process can lead to samples weighted to those species most readily detected and biased estimates of community composition [40], [41], [42].

## Results

We found that inference about treatment effects on the avian community would be consistent regardless of which set of priors we considered, although we note that the effect was positive in all 4 cases (Table 1). The analysis with informed detection and treatment priors provided estimates with similar precision to those from the uninformative analysis for most quantities of interest (Figure S1, 1–7). Also, the population-level hyper-parameter for the treatment effect in this analysis was smaller than that from the uninformative analysis. As a result, we used this analysis for inference and discussion.

**Table 1:** Population-level hyper-parameter (95% credible intervals) for effect of salvage-logging of beetle-killed stands on avian occupancy rates (α), Fremont and Winema National Forests, south-central, Oregon, USA, 1996–1998.

Priors Used	Posterior Median	95% Credibility Interval
Diffuse	1.48	−0.11, 3.44
Prior on Detection only	0.75	−0.41, 2.05
Prior on Treatment only	0.62	−0.49, 2.05
Prior on Detection and Treatment	1.04	−0.14, 2.51

A positive estimate indicates that occupancy was greater on harvested plots and a negative estimate indicates that occupancy was less on harvested plots. We considered 4 scenarios: 1.) detection and occupancy priors were diffuse; 2.) detection intercept priors informed by prior information; 3.) occupancy priors (the treatment effect) informed by prior information; and 4.) both detection intercept and occupancy treatment effect priors informed by prior information.

**Figure 1:**
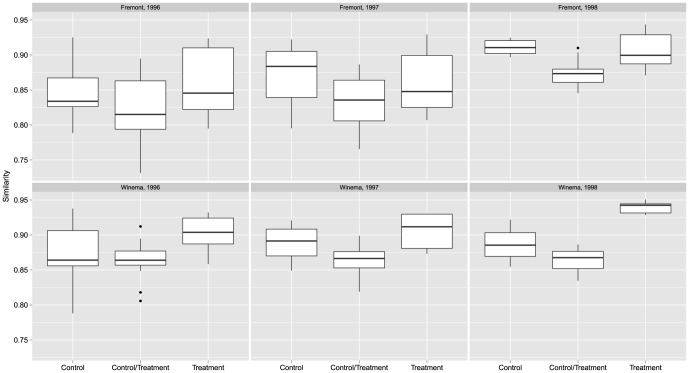
Boxplot summaries of median species similarity among control, control and treatment, and treatment plots, Fremont and Winema National Forests, south-central Oregon, USA, 1996–1998.

**Figure 2:**
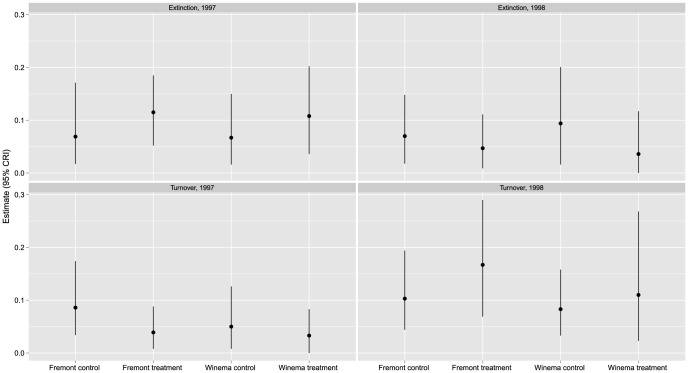
Estimates of turnover and extinction rates on 12 control and 12 treatment plots, Fremont and Winema National Forests, south-central, Oregon, USA, 1996–1998. Turnover is the probability that a species selected at random from community at time *t* is a species not present at time *t*–1. Extinction is the probability that a species that occupied a plot at time *t* did not occupy the plot in time *t* +1.

On average, treatment plots contained an estimated 0.2–2.2 more species than control plots (based on the estimated difference between treatment and control plots; Table 2); however, in all cases the 95% credible intervals included 0. Median similarity between control and treatment stands ranged from 0.82–0.87 (Figure 1). In general, treatment and control plots on Winema were more similar in species richness than plots on Fremont. Median similarity ranged from 0.83–0.91 among control plots (Figure 1) and from 0.85–0.94 among treatment plots (Figure 1). Turnover and extinction rates were similar on control and treatment plots in both districts (Figure 2). Turnover rates appeared to increase in 1998 on both districts (Figure 2). Similarly, extinction rates were slightly lower on both districts in 1998 than in 1997 (Figure 2). The most commonly detected of the 27 species included in the analysis were Mountain Chickadee, Yellow-rumped Warbler, Dark-eyed Junco, Chipping Sparrow, Dusky Flycatcher, American Robin, Hermit Thrush, and Cassin's Finch (Table S1).

**Table 2:** Treatment contrasts for species richness on 12 treatment and 12 control plots, Fremont and Winema National Forests, south-central Oregon, USA, 1996–1998.

Treatment Contrast	Posterior Median	95% Credibility Interval
Fremont 1996	2.17	−0.33, 4
Fremont 1997	0.17	−2.00, 2.33
Fremont 1998	1.67	−0.50, 4.17
Winema 1996	1.58	−1.17, 3.58
Winema 1997	0.17	−2.25, 2.33
Winema 1998	1.83	−0.83, 4.67

We estimated the mean species richness for the four treatment × district combinations for each MCMC sample and then subtracted them to create treatment contrasts.

**Figure 3:**
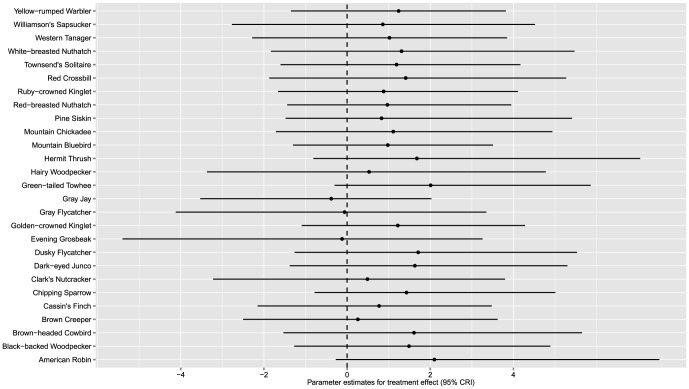
Parameter estimates (α) and 95% credible intervals for the effect of salvage-logging of beetle-killed stands on occupancy rates for 27 avian species detected on 24 study plots on Fremont and Winema National Forests, south-central, Oregon, USA, 1996-1998. Species with estimates below 0 declined on harvested plots while species with estimates above 0 increased on harvested plots.

We did not find any evidence for an effect of beetle-killed forest stands on occupancy rates for any of the 27 breeding species (i.e., 95% credible intervals included 0 for all species; Figure 3) using any of the prior distributions. Twenty-four of the species (90%) showed a positive mean response to the treatment and 5 species had a negative mean response (Figure 3). Among cavity-nesting species, both primary (Williamson's Sapsucker, Black-backed, and Hairy Woodpeckers) and secondary cavity nesters (Mountain Bluebird, White-breasted Nuthatch, Red-breasted Nuthatch, and Mountain Chickadee) responded positively (Figure 3).

**Figure 4:**
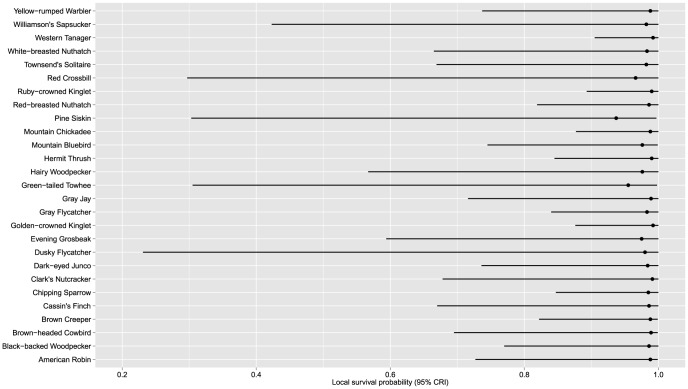
Estimates of local survival probability (probability that a species that occurred on a plot in one year also occurred on that plot the previous year) and 95% credible intervals for 27 avian species detected on 24 study plots on Fremont and Winema National Forests, south-central, Oregon, USA, 1996–1998.

Estimates of local survival probability were high for all species, although estimates were imprecise for Evening Grosbeak, Pine Siskin, Red Crossbill, Green-tailed Towhee, Dusky Flycatcher, Hairy Woodpecker, and Williamson's Sapsucker (Figure 4). Average local survival ranged from 0.93–1.0, indicating that species were likely to occur on the same plots across the 3 years in which we sampled.

**Figure 5:**
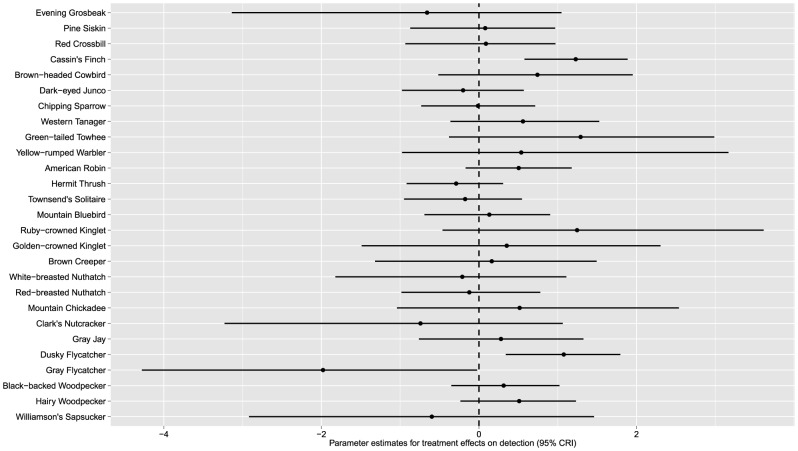
Parameter estimates and 95% credible intervals for the effect of salvage-logging beetle-killed stands (β) on detection rates for 27 avian species detected on 24 study plots on Fremont and Winema National Forests, south-central, Oregon, USA, 1996–1998. Species with estimates below 0 were less likely to be detected on harvested plots while species with estimates above 0 were more likely to be detected on harvested plots.

We did find evidence for an effect of harvesting beetle-killed forest stands (i.e., 95% credible intervals did not include 0) on detection rates for some of the species (Figure 5). We found evidence of a positive treatment effect on detection for Cassin's Finch and Dusky Flycatcher and a negative effect on Gray Flycatcher. Among cavity-nesting species, White-breasted Nuthatch, Red-breasted Nuthatch, and Williamson's Sapsucker was less likely to be detected in treatment than in control stands (Figure 5).

## Discussion

Management interventions implemented after natural disturbances seek to recoup economic value from affected areas or to reduce the risk of subsequent disturbances. Prescriptions can have non-target effects that reduce habitat quality and alter species distributions and community composition. We did not find any evidence that pay-as-cut salvage logging prescriptions in beetle-killed lodgepole pine forests had an impact on avian species composition. These findings stand in contrast to studies that investigated avian responses to salvage logging (often clearcutting) of forests burned by wildfire (so-called post-fire forests), which often find significant deleterious effects on the avian community [43], [44], [45].

Significant uncertainty remains about viable management alternatives for forests impacted by mountain pine beetle outbreaks [14], [46], [47], [48], [49]. These debates are complicated by potential changes to outbreak dynamics as a result of regional climate change [13] and management prescriptions [50]. Our results suggest that a carefully monitored harvest prescription can reduce potential fuel loads [5] in beetle-killed forests without changing avian species richness or reducing occupancy rates of individual species. We suspect that our results arise from the comparatively less severe effects on habitat structure of both the beetle outbreak and the pay-as-cut salvage logging, as opposed to clearcut salvage logging following other disturbance types [6], [51], [52]. For example, the beetle outbreak affected live trees and did little damage to other habitat features, such as understory shrubs. In contrast, fire and tornadoes have the potential to alter all habitat features in a stand. Similarly, the pay-as-cut prescription reduced snag numbers in treatment stands but did not result in substantial changes to other habitat features such as canopy cover, understory shrub cover, or the number of live trees (Table S2). Clearcutting will remove most of the standing wood volume, including both live and dead trees, and has the potential to affect other habitat features [53], [54]. In general, the pay-as-cut prescription is best considered as a type of structural retention harvesting that led to only minor changes in forest structure [31], [55].

Our results concur with other studies of avian community responses to management interventions in eastside forests of the Cascade Mountains in Oregon and Washington, USA. Generally, these studies have found no effect or mixed effects (some species decrease in number while others increase) of prescribed fire or salvage-logging [17], [56], [57]. However, an important caveat from all of these studies, as well as the present study, is that study designs and sampling techniques may be inadequate to capture responses of species most likely to be affected negatively by interventions. For example, species-specific studies for strong cavity nesters that occur at low population densities (black-backed and hairy woodpeckers) may be required to derive strong inference about how these species respond to both natural and anthropogenic disturbances. Researchers can capitalize on modern analytical techniques, such as multilevel models, to estimate responses [42], [57], although these techniques might not accurately capture responses of species with large home ranges that likely use habitat outside of sample plots. In these cases, radio-telemetry studies are more likely to provide reliable information about habitat- and treatment-specific responses [58]. Finally, when prior information suggests that certain species are more likely to be impacted than others, evaluation of community responses may not be the most efficient allocation of limited resources.

The ability to incorporate prior information into current investigations is an oft-cited advantage of Bayesian statistical methods [59], [60], [61]. Incorporating information from previously completed studies offers practical advantages such as increasing precision of parameter estimates [62]. In addition, priors can be used to represent different opinions about magnitude and direction of a proposed effect. For example, we thought that informing priors with results from a management prescription in a similar study system was reasonable, and this decision led to a positive treatment effect (the diffuse prior led to a positive treatment effect, too). Others may think that a negative effect was justified. Their belief could be formalized in the prior, and the posterior distributions would then reflect this prior belief and the observed data. We did not find any evidence of differences among treatment effects in the four analyses that we conducted with different priors, and considered using the analysis with informed detection and treatment priors to be a reasonable decision. Despite the increasing prominence of Bayesian methods in ecology and other disciplines, researchers can find little guidance about the basis for selecting information or how to include information in complicated analyses. For example, how similar should the ecological and/or management context be between studies? Should informed priors be used for both observation and state parameters in multilevel models? Which analysis, among several based on different priors, should be the basis for inference? Researchers may be challenged to make an objective decision in those cases where inference does change across analyses. At the minimum, we suggest that an analysis with diffuse priors be included alongside an analysis with informed priors. Finally, many researchers may consider using informed priors for the observation process to be reasonable, given that this portion of the model is not of primary interest. Even this decision may have important consequences for inference, though, given the way in which the observation and state processes are often conflated [42].

Land use intensification plays a critical role in provisioning rapidly growing human populations [63], [64], [65]. However, managers must balance economic gains from prescriptions against potentially severe consequences for maintenance of native biological diversity [66]. Large-scale field experiments that evaluate operational prescriptions provide insight into potential trade-offs between commercial extraction of resources and conservation of wildlife communities after natural disturbances. Species richness is frequently measured in research studies and management programs to assess community responses to anthropogenic and natural disturbances [67], [68]. However, species occupancy (e.g., at the stand level) may remain unchanged even if demographic measures such as survival and reproduction vary, a critical result for management of individual populations. Our results suggest that salvage-logging of beetle-killed stands did not exert an effect on avian community composition. Taken together with additional analyses of avian abundance and nest survival from this same study [69], [70], these results suggest that a carefully monitored harvest prescriptions can reduce potential fuel loads without changing avian species richness or reducing occupancy rates of individual species. As in most cases, longer term monitoring of population dynamics is prudent to identify negative responses, and the causes generating them, if they arise.

## Methods

### 1. Study Area & Treatment Descriptions

The Fremont and Winema National Forests (FR and WI) occur in south-central Oregon, USA (43° N, 122° W). These management units lie within the Central Oregon Pumice Zone, an area dominated by xeric forests of lodgepole (*Pinus contorta*) and ponderosa pine (*P. ponderosa*) [71]. Elevations range from 1585–1951 m. The climate is characterized by lower summer rainfall, large diurnal temperature fluctuations, and a truncated growing season [72]. Historical disturbance regimes included both fires and beetle outbreaks [73]. Mountain pine beetle (*Dendroctonus ponderosae*) epidemics that occurred in the 1980's resulted in extensive mortality over ∼250,000 ha [73], [74].

Study sites occurred within 2 timber sale areas: the Comet/Cupid Timber Sale area on the Silver Lake Ranger District of the FR and the First Timber Sale Area on the Chemult Ranger District of the WI. The harvest prescription applied to treatment stands is referred to as “pay-as-cut”: the operator paid only for the wood volume removed from the sale area, rather than having purchased a fixed volume of wood. In the latter scenario, financial incentives exist to remove as much wood as possible; in the former, operators tend to focus on areas with high concentrations of accessible volume (in this case, snags) to minimize operational expenses. Within the boundary of the harvest stands, all dead standing or down lodgepole pine were available for harvest. Live trees of all species, dead ponderosa pine, and partially dead (dead tops) trees were not available for harvest. Downed logs were retained according to Forest Plan Standards and Guidelines (25 pieces >2.4 m in length and 15.2 cm in diameter at the large end per ha) [75], [76] and slash was piled at landings throughout the sale area. As a consequence of these guidelines, our results reflect variation under operational conditions (rather than having treatment levels monitored by investigators). Harvesting was completed on the FR in 1994 and on the WI by spring 1996.

### 2. Study Design and Field Data Collection

Within both areas, we used United States Department of Agriculture-Forest Service timber stand boundaries to define discrete stands available for inclusion in the study. Our only requirement was that stands were ≥25 ha in size and relatively uniform with regard to tree species composition and age. Managers allocated stands as either treatment (harvested with pay-as-cut) or control stands. A total of 9 control and 21 treatment stands were available for sampling on the FR; a total of 11 control and 7 treatment stands were available for sampling on the WI. We randomly selected 6 control and 6 treatment stands from the pool of available stands for both FR and WI, resulting in 24 total stands in the study. Average stand size was 201 ha. We randomly placed 8 point count stations within each sample stand, regardless of stand size, to sample avian species richness. We used a 250 m grid placed on stand maps and a random number table to allocate point count stations randomly within each stand. The grid was sized so that point count stations were >100 m from the edge of the stand (to prevent sampling of birds from adjacent stands). The minimum distance between point count stations was 250 m, but in some cases the distance was much greater than 750 m. We received written or verbal permission to sample sites from all public landowners involved in the study. No formal permits were required.

We used a standardized point count protocol [77] to sample birds 3 times per year from 15 May to 1 July. At each point count station, we recorded all birds seen or heard within 50 m of the station during a 5 minute sampling period. Surveys began at sunrise and ended within 4 hours after sunrise. We sampled stations in random order during each session. Two experienced observers conducted the bird surveys during each year of the survey in order to reduce potential observer bias [77], [78]. We conducted point count surveys from 1996–1998 (post-treatment) on all 24 stands.

We measured habitat variables within 11.3-m radius plots [79] randomly placed within each stand (n = 32 per stand). We counted the total number of snags and live trees >10 cm dbh. We measured average overstory canopy coverage with a Moosehorn cover scope [80] at 20 points within each plot. We estimated percent shrub cover (all shrubs regardless of size) by dividing each plot into quarters and summing across the whole plot. As expected, treatment plots contained fewer snags, on average, than control stands once harvesting was completed [70]. However, the number of live trees, percent canopy cover, and percent cover of shrubs did not vary substantially between treatment and control stands (Table S2).

### 3. Model Construction

We used a multispecies site occupancy model [42], [81] to estimate species level treatment effects as well as population level summaries of occupancy, such as species richness and species similarity, between treatment and control stands [81], [82]. In addition, we estimated occupancy dynamics, including species turnover, extinction, and survival probability [57]. We estimated occupancy for those species that are known to be present as breeding populations in study stands [69]. Following Russell et al. [57], we do not account for the contribution of unobserved species in our population estimates, instead conditioning on the set of observed breeding species in our study. We let 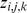 denote true the occupancy status, in which 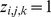 if species 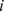 occupies site 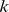 for year 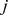 or 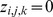 otherwise. The occupancy state is taken to be a Bernoulli random variable,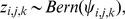 where 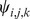 is the probability that species 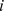 occupies site 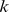 for year 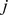. We take species detection to again follow a Bernoulli distribution, 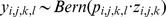, where 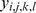 is 1 if the species 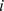 is detected at site 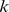 during year 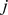, during visit 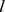, or 0 otherwise and where 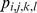 is the detection probability. Note that under this parameterization, the probability of detecting the species 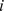 at site 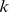 and year 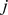 will be zero if it does not occupy site 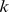, since 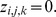


We considered the model based on the experimental design, in which detection probability varied by treatment type (either control or harvested) and year. In addition, we included linear and quadratic terms for Julian date (January 1 = 1, December 31 = 365) because avian detection rates are known to vary seasonally [83] We centered and scaled the date covariate. The species-specific detection probability mean model is: 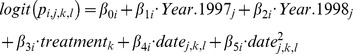



Occupancy was allowed to vary by management district and by an interaction of treatment type and year. The occupancy mean model for the year 1996 is: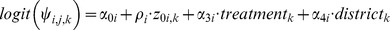 and the mean model for years 1997 and 1998:
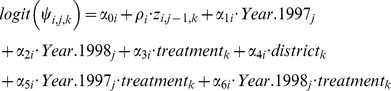



Note that 1996 is the base year for the dynamic model. The terms 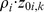 and 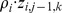 allow the occupancy of site 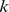 and year 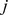 to be related to the occupancy in the previous year [36], [57]. The remaining terms are indicator variables for treatment, district, and year.

Under the hierarchical community model, we assume that the species-specific effects for a given parameter are drawn from a common normal distribution, e.g., that 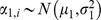
for parameter 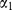
of species *i*, where the mean and variance of 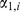 are population-level hyper-parameters. This population-level distribution provides a summary of community response, both in terms of the mean behavior as well as the variability in behavior. The extent to which information is shared across species depends on both the degree of uniformity across the population, as estimated by the population-level parameters, and the amount of information available for each species. For species with little information, those with low detection probabilities, estimates will tend to shrink toward the population mean value. Priors used in the analysis are in Appendix 5, in Text S1.

We estimated species richness for treatment and control plots separately as: 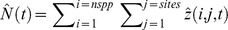 where *nspp* is the total number of species across all sites. To examine the affect of pay-as-cut logging on species richness, we estimated the mean species richness for the four treatment × district combinations for each MCMC sample and then subtracted them to create contrasts. We then calculated posterior medians and 95% credible intervals. These contrasts can be used to determine if the number of species that are on treatment stands is different than on control stands. In addition to estimated species richness, we estimated species similarity both between and among treatment and control stands [81] by calculating the proportion of species that occupy both stands. Species similarity in year *j* for stands 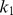 and 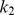, is defined as:
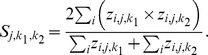



Within each year, we estimated the similarity for all pairwise combinations of stands. This set of summary statistics allows us to determine the impact of salvage logging on species similarity.

We estimated species turnover (τ), the probability that a species chosen at random from the community at time *j* is a species not present at time *j* – 1 and local-extinction rates (ε) as:
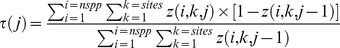


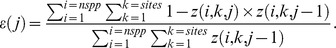



An oft-cited strength of Bayesian methods is the ability to incorporate prior information directly into analyses [59], [84]. Doing so can yield more precise estimates of quantities of interest and associated measures such as credible intervals [62]. We used estimates from Russell et al. [57] to provide prior information for species-level detection and occupancy probabilities. Our justification was three-fold. First, avian communities were similar, with 26/27 (96%) species (we used information from the spotted towhee *Pipilo maculates* for the green-tailed towhee *Pipilo chlorurus*) in our study also occurring in the study area sampled by Russell et al. (2009). Second, both studies used similar sampling (point-count stations) and analytical tools (hierarchical models that accounted for variable detection and which treated species as random effects). Third, other studies have found that avian communities are resilient to natural [85], [86] and management [17], [87] disturbances of moderate intensity. Russell et al. [57] examined avian responses to prescribed understory burns, a comparatively mild form of disturbance use to reduce fuel loads [5], in a dry forest environment in the eastern Cascade Mountains, USA. The mechanical treatment in our study was similarly designed to have a small impact on the forest environment while reducing fuel loads and was also located in the eastern Cascade Mountains, approximately 500 miles south of where the Russell et al. [57] study occurred.

We evaluated effects of incorporating prior information by comparing results from models using both diffuse and informative priors. Although the model contains many priors, we only considered using prior information for the hyperprior mean for the detection probability intercept and occupancy treatment effect. We considered 4 scenarios: 1.) all priors were diffuse; 2.) detection priors are informed by prior information; 3.) occupancy priors (the treatment effect) are informed by prior information; and 4.) both detection and occupancy priors are informed by prior information. In the analysis with diffuse priors (Scenario 1), we used a 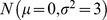 prior for the detection intercept and a 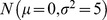 prior for the occupancy treatment contrast. In the informed analyses, we used a 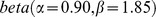 prior for the detection intercept (Scenarios 2, 4) and 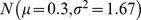 for the occupancy treatment contrast (Scenarios 3, 4).

To obtain the informative priors for the analysis, we used posterior summaries for all species in our study that overlapped with Russell et al. [57]. We created histograms of the species-specific posterior means for the detection probability intercept and the occupancy treatment contrast. We then fit two distributions, a beta for detection and a normal for the treatment effect on occupancy, to the histograms (Figure S2). The estimated distributions are the informed hyper priors for the detection probability intercept and the treatment effect on occupancy in our analysis.

### 4. Model Fitting and Analysis

We fit our model using WinBUGS [88] called from R (R Development Core Team 2010) using the ‘bugs’ function in package R2WinBUGS [89], [90]. For all models, we ran 3 Markov chains of length 500,000 with a burn-in period of 250,000 and 1/50 thinning. We assessed convergence using the Gelman-Rubin statistic [62] and visual inspection of the chains, with both measures indicating a reasonable assumption of convergence. We provide all code for this model in Text S1. To assess consistency between our models and data, we used posterior predictive checks [62]. We did not find any evidence of lack of fit in the models that we evaluated. We provide details and an example for the posterior predictive checks in Text S2.

## Supporting Information

Figure S1 **1–7: Results of analyses using four different sets of priors to evaluate avian community responses to salvage-logging of beetle-killed forests, Fremont and Winema National Forests, south-central Oregon, USA, 1996–1998.** We considered 4 scenarios: 1.) both priors were diffuse; 2.) detection intercept priors informed by prior information; 3.) occupancy priors (the treatment effect) informed by prior information; and 4.) both detection and occupancy priors informed by prior information.
(EPS)

Figure S2 **Histograms of species-specific posterior means for the detection probability intercept and the occupancy treatment contrast for the 27 avian species on control and salvage logged plots in beetle-killed lodgepole pine forests, Fremont and Winema National Forests, south-central Oregon, USA, 1996–1998.**
(PDF)

Table S1 **Number of individual detections of 27 avian species on control and salvage logged plots in beetle-killed lodgepole pine forests, Fremont and Winema National Forests, south-central Oregon, USA, 1996–1998.**
(PDF)

Table S2 **Summary statistics for 4 habitat characteristics by treatment type and district, Fremont and Winema National Forests, south-central Oregon, USA, 1996–1998.**
(PDF)

Text S1 **WinBUGS code for hierarchical community model to evaluate avian community responses of salvage-logging of beetle-killed lodgepole pine forests, Fremont and Winema National Forests, south-central Oregon, USA, 1996–1998.**
(PDF)

Text S2 **Posterior predictive checks to assess goodness of fit for Bayesian models, Fremont and Winema National Forests, south-central Oregon, USA, 1996–1998.**
(PDF)
