# Supplementary material for: Effects of Management Intervention on Post-Disturbance Community Composition: An Experimental Analysis Using Bayesian Hierarchical Models
Source: PLoS One. 2013 Mar 22;8(3):e59900. doi: 10.1371/journal.pone.0059900 (PMC3606292; doi:10.1371/journal.pone.0059900)

Figure S4: Determining Informative Priors

Histograms of species-specific posterior means for the detection probability intercept and the occupancy treatment contrast for the 27 avian species on control and salvage logged plots in beetle-killed lodgepole pine forests, Fremont and Winema National Forests, south-central Oregon, USA, 1996–1998. Posterior means were taken from Russell et al. (2009). We fit two distributions to these data, a beta for detection and a normal for the treatment effect on occupancy. The estimated distributions are the informed hyper priors in our analyses. The dashed lines are the uninformative and the solid are based on Russell's.

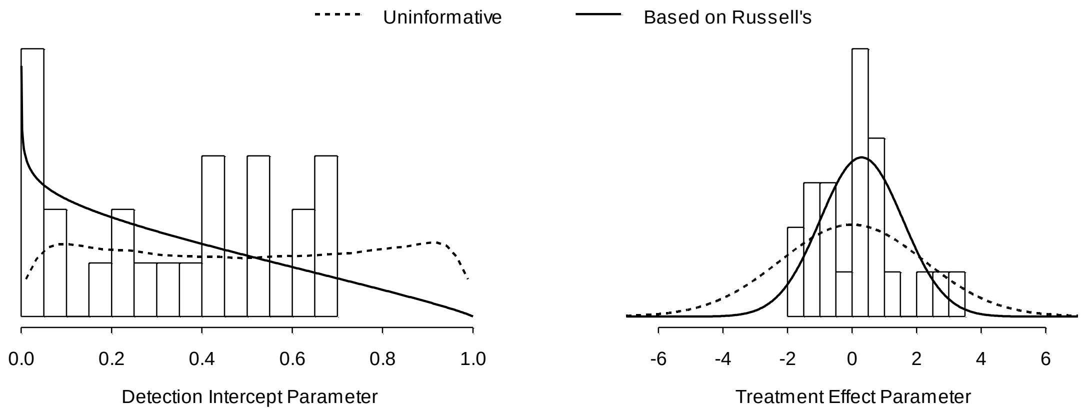

Supplement: Figure S2 — Histograms of species-specific posterior means for the detection probability intercept and the occupancy treatment contrast for the 27 avian species on control and salvage logged plots in beetle-killed lodgepole pine forests, Fremont and Winema National Forests, south-central Oregon, USA, 1996–1998. (PDF) [file pone.0059900.s002.pdf]
