# Supplementary material for: Effects of Management Intervention on Post-Disturbance Community Composition: An Experimental Analysis Using Bayesian Hierarchical Models
Source: PLoS One. 2013 Mar 22;8(3):e59900. doi: 10.1371/journal.pone.0059900 (PMC3606292; doi:10.1371/journal.pone.0059900)
Supplement: Table S1 — Number of individual detections of 27 avian species on control and salvage logged plots in beetle-killed lodgepole pine forests, Fremont and Winema National Forests, south-central Oregon, USA, 1996–1998. (PDF) [file pone.0059900.s003.pdf]

Appendix S2: Number of detections by species in control and treatment stands, south-central Oregon, USA, 1996-1998.

Species		Code	Guild	Control	Treatment	Total	AOU order
Williamson's Sapsucker	*Sphyrapicus thyroideus*	WISA	Aerial insectivore	3	6	9	1
Hairy Woodpecker	*Picoides villosus*	HAWO	Bark insectivore	37	58	95	2
Black-backed Woodpecker	*Picoides arcticus*	BBWO	Bark insectivore	52	49	101	3
Gray Flycatcher	*Empidonax wrightii*	GRFL	Aerial insectivore	64	9	73	4
Dusky Flycatcher	*Empidonax oberholseri*	DUFL	Aerial insectivore	157	178	335	5
Gray Jay	*Perisoreus candensis*	GRJA	Generalist	43	17	60	6
Clark's Nutcracker	*Nucifraga columbiana*	CLNU	Specialist seed	10	0	10	7
Mountain Chickadee	*Poecile gambeli*	MOCH	Foliage gleaning	790	700	1490	8
Ruby-crowned Kinglet	*Regulus calendula*	RCKI	Specialist seed	1	12	13	9
Western Tanager	*Piranga ludoviciana*	WETA	Foliage gleaning	15	39	54	10
Brown-headed Cowbird	*Molothrus ater*	BHCO	General ground	14	28	42	11
Golden-crowned Kinglet	*Regulus satrapa*	GCKI	Foliage gleaning	0	5	5	12
Red Crossbill	*Loxia curvirostra*	RECR	Bark insectivore	29	36	65	13
Mountain Bluebird	*Sialia currucoides*	MOBL	Aerial insectivore	38	53	91	14
Townsend's Solitaire	*Myadestes townsendi*	TOSO	Foliage gleaning	42	25	67	15
Hermit Thrush	*Catharus guttatus*	HETH	Foliage gleaning	121	102	223	16
American Robin	*Turdus migratorius*	AMRO	Generalist	148	187	335	17
Yellow-rumped Warbler	*Dendroica coronata*	YRWA	Foliage gleaning	695	606	1301	18
Green-tailed Towhee	*ilo chlorurus*	GTTO	Seeds/insects	3	25	28	19
White-breasted Nuthatch	*Sitta carolinensis*	WBNU	Specialist seed	10	6	16	20
Chipping Sparrow	*Spizella passerina*	CHSP	Seeds/insects	318	291	609	21
Dark-eyed Junco	*Junco hyemalis*	DEJU	Seeds/insects	369	338	707	22
Brown Creeper	*Certhia americana*	BRCR	Bark insectivore	10	15	25	23
Cassin's Finch	*Carpodacus cassinii*	CAFI	Seeds/insects	59	143	202	24
Red-breasted Nuthatch	*Sitta canadensis*	RBNU	Aerial insectivore	43	30	73	25
Pine Siskin	*Carduelis pinus*	PISI	General ground	104	91	195	26
Evening Grosbeak	*Coccothraustes vespertinus*	EVGR	Specialist seed	11	0	11	27
TOTAL				3186	3049	6235	
