# Supplementary material for: Effects of Management Intervention on Post-Disturbance Community Composition: An Experimental Analysis Using Bayesian Hierarchical Models
Source: PLoS One. 2013 Mar 22;8(3):e59900. doi: 10.1371/journal.pone.0059900 (PMC3606292; doi:10.1371/journal.pone.0059900)
Supplement: Table S2 — Summary statistics for 4 habitat characteristics by treatment type and district, Fremont and Winema National Forests, south-central Oregon, USA, 1996–1998. (PDF) [file pone.0059900.s004.pdf]

Appendix S3: Average and 95% confidence interval for 4 habitat variables by management district and treatment type, south-central Oregon, USA, 1996–1998. Summary statistics were calculated for each stand based on 4 11.3m (0.04 ha) radius sampling plots located within a 50 m circle and centered on each point count station (*n* = 8 per stand). For each district and treatment type combination, *n* = 48.

District and treatment	Snags > 10 cm dbh	95% CL	Trees > 10 cm dbh	95% CL	Canopy cover	95% CL	Shrub cover	95% CL
Fremont Control	1.6	1.1 − 2.1	6.6	5.2 − 8	16	15 − 18	5	2 − 7
Fremont Treatment	0.2	0.1 − 0.3	5.8	4.5 − 7.2	12	10 − 14	4	3 − 6
Winema Control	2.3	1.6 − 3.1	7.7	6.1 − 9.3	13	11 − 15	12	10 − 14
Winema Treatment	0.9	0.3 − 1.5	8.4	6.7 − 10	10	9 − 12	9	7 − 11
