# Supplementary material for: Effects of Management Intervention on Post-Disturbance Community Composition: An Experimental Analysis Using Bayesian Hierarchical Models
Source: PLoS One. 2013 Mar 22;8(3):e59900. doi: 10.1371/journal.pone.0059900 (PMC3606292; doi:10.1371/journal.pone.0059900)
Supplement: Text S1 — WinBUGS code for hierarchical community model to evaluate avian community responses of salvage-logging of beetle-killed lodgepole pine forests, Fremont and Winema National Forests, south-central Oregon, USA, 1996–1998. (PDF) [file pone.0059900.s005.pdf]

Appendix S5: WinBUGS code for hierarchical community model to evaluate avian community responses of salvage-logging of beetle-killed lodgepole pine forests, Fremont and Winema National Forests, south-central Oregon, USA, 1996–1998.

```
# --------------------------------------------------------------------------
# Load necessary libraries
# --------------------------------------------------------------------------

library(R2WinBUGS)
library(coda)
library(reshape)

# --------------------------------------------------------------------------
# read in the data and package for WinBUGS
# --------------------------------------------------------------------------


dat0 <- read.csv("D:\\Giovanini data\\Timberlands\\Enviromental Research\\Chemult
Study\\data\\Community Occupancy Chemult data.csv")


# removing the pre-treatment year
dat <- dat0[dat0$year>1995,]

# calculating the observed site level occupancy
dat$occ <- with(dat, ifelse(nL50>0, 1,0))


dat1996 <- dat[dat$year==1996,]
dat1997 <- dat[dat$year==1997,]
dat1998 <- dat[dat$year==1998,]


# Creating array of occupancy


temp.melt <- melt(dat, id.var=c("spcode", "year", "stand", "visit"), measure.var="occ")
X <- cast(temp.melt, visit~ stand~ year ~spcode)
rm(temp.melt)


nsite <-  c(22, 22, 22) # c(22, 24, 24) 22 SITES SINCE CAN'T DO LOCAL SURVIVAL FOR SITES 23 & 24

nspecies <- dim(X)[4]
nyear <- dim(X)[3]



nvisit.start.1996 <- c(rep(1, nsite[1]))
nvisit.start.1997 <- rep(1, nsite[2])
nvisit.start.1998 <- c(rep(1, 9), 2, rep(1,12))

nvisit.start <- matrix(c(nvisit.start.1996, nvisit.start.1997, nvisit.start.1998), byrow=T,
       nrow=3)


nvisit <- 3


trt <- as.numeric(unlist(lapply(split(ifelse(dat1997$trt=="C", 0, 1),
       as.character(dat1997$stand)), min)))

district <- as.numeric(unlist(lapply(split(ifelse(dat1997$district=="F", 0, 1),
       as.character(dat1997$stand)), min)))


Winema.trt <- as.numeric(unlist(lapply(split(ifelse(dat1997$district=="W" &  dat1997$trt=="T", 1,
       0), as.character(dat1997$stand)), min)))
Winema.cont <- as.numeric(unlist(lapply(split(ifelse(dat1997$district=="W" &  dat1997$trt=="C",
```

```
        1, 0), as.character(dat1997$stand)), min)))

Fremont.trt <- as.numeric(unlist(lapply(split(ifelse(dat1997$district=="F" &  dat1997$trt=="T",
        1, 0), as.character(dat1997$stand)), min)))
Fremont.cont <- as.numeric(unlist(lapply(split(ifelse(dat1997$district=="F" &  dat1997$trt=="C",
        1, 0), as.character(dat1997$stand)), min)))
year.1996 <- c(1,0,0)
year.1997 <- c(0,1,0)
year.1998 <- c(0,0,1)


# ---------------------------------------------------------------------------
# read in visit data
# ---------------------------------------------------------------------------



visit.data0 <- read.csv("D:\\Giovanini data\\Timberlands\\Enviromental Research\\Chemult
Study\\data\\Chemult Stand visit data.csv")

visit.data <- visit.data0[ visit.data0$year>1995,]

# Centering and scaling date
visit.data$Centered.Scaled.Date <- with(visit.data, (StandDateJ-mean(StandDateJ))/sd(StandDateJ))


temp.melt.date <- melt(visit.data, id.var=c("year", "stand", "visit"),
        measure.var="Centered.Scaled.Date")
Julian.date.mat <- cast(temp.melt.date, visit~ stand~ year)
rm(temp.melt.date)


# ----------------------
# 2. Specify the models
# ----------------------


chemult.occ.model  <- function(){

        # Prior distributions for community-level parameters
                                pi0 ~dunif(0, 1)
                                mu.a.0 ~dnorm(0, 0.333)
                                mu.rho ~dnorm(0, 0.333)

                                mu.a.1997 ~dnorm(0, 0.2)
                                mu.a.1998 ~dnorm(0, 0.2)
                                mu.a.trt  ~dnorm(.3, 0.60)  # Diffuse prior for
                                                            # mu.a.trt was dnorm(0, 0.2)
                                mu.a.Winema ~dnorm(0, 0.2)
                                mu.a.1997.trt ~dnorm(0, 0.2)
                                mu.a.1998.trt ~dnorm(0, 0.2)

                                                    # Diffuse prior was mu.b.0 ~dnorm(0, 0.333)

                                detection.prob.int ~dbeta(0.90, 1.85)
                                mu.b.0 <-log(detection.prob.int/(1-detection.prob.int))


                                mu.b.1997 ~dnorm(0, 0.2)
                                mu.b.1998 ~dnorm(0, 0.2)
                                mu.b.trt  ~dnorm(0, 0.2)

                                mu.b.date  ~dnorm(0, 0.2)
                                mu.b.date.2 ~dnorm(0, 0.2)



                                sig2.a.0  ~dgamma(2, .5)
                                sig2.rho  ~dgamma(2, .5)
                                sig2.a.1997 ~dgamma(2, .5)
                                sig2.a.1998 ~dgamma(2, .5)
```

```
                                sig2.a.trt  ~dgamma(2, .5)
                                sig2.a.Winema ~dgamma(2, .5)
                                sig2.a.1997.trt ~dgamma(2, .5)
                                sig2.a.1998.trt ~dgamma(2, .5)

                                sig2.b.0 ~dgamma(2, .5)
                                sig2.b.1997 ~dgamma(2, .5)
                                sig2.b.1998 ~dgamma(2, .5)
                                sig2.b.trt  ~dgamma(2, .5)
                                sig2.b.date ~dgamma(2, .5)
                                sig2.b.date.2 ~dgamma(2, .5)

                                tau.a.0 <- 1/ sig2.a.0
                                tau.rho <- 1/ sig2.rho
                                tau.a.1997 <- 1/sig2.a.1997
                                tau.a.1998 <- 1/sig2.a.1998
                                tau.a.trt <- 1/sig2.a.trt
                                tau.a.Winema <- 1/sig2.a.Winema
                                tau.a.1997.trt <- 1/sig2.a.1997.trt
                                tau.a.1998.trt <- 1/sig2.a.1998.trt

                                tau.b.0 <- 1/sig2.b.0
                                tau.b.1997 <- 1/sig2.b.1997
                                tau.b.1998 <- 1/sig2.b.1998
                                tau.b.trt <- 1/sig2.b.trt


                                tau.b.date <- 1/sig2.b.date
                                tau.b.date.2 <- 1/sig2.b.date.2

# Species level loop
                for (i in 1:nspecies) {

   #Prior distributions for the occupancy and detection covariates for each species


                                a.0[i] ~dnorm(mu.a.0, tau.a.0)
                                rho[i] ~dnorm(mu.rho, tau.rho)
                                a.1997[i] ~dnorm(mu.a.1997, tau.a.1997)
                                a.1998[i] ~dnorm(mu.a.1998, tau.a.1998)
                                a.trt[i]  ~dnorm(mu.a.trt, tau.a.trt)
                                a.Winema[i] ~dnorm(mu.a.Winema, tau.a.Winema)
                                a.1997.trt[i] ~dnorm(mu.a.1997.trt, tau.a.1997.trt)
                                a.1998.trt[i] ~dnorm(mu.a.1998.trt, tau.a.1998.trt)


                                b.0[i] ~dnorm(mu.b.0, tau.b.0)
                                b.1997[i] ~dnorm(mu.b.1997, tau.b.1997)
                                b.1998[i] ~dnorm(mu.b.1998, tau.b.1998)
                                b.trt[i]  ~dnorm(mu.b.trt, tau.b.trt)

                                b.date[i] ~dnorm(mu.b.date, tau.b.date)
                                b.date.2[i]  ~dnorm(mu.b.date.2, tau.b.date.2)

           # Year 1996 Model loop
             for (j in 1:1){

              # Create a loop to estimate the Z matrix (true occurrence for species i at stand j.
                    for (k in 1:nsite[j]) {

                      z0[i,k] ~ dbern(pi0)


logit(psi[k,j,i]) <-  a.0[i] + rho[i]*z0[i,k] + a.1997[i]*year.1997[j] + a.1998[i]*year.1998[j] +
       a.trt[i]*trt[k]  + a.Winema[i]*district[k]+ a.1997.trt[i]*year.1997[j]*trt[k] +
       a.1998.trt[i]*year.1998[j]*trt[k]

mu.psi[k,j,i] <- min(0.99, max(psi[k,j,i], 0.01))
Z[k,j,i] ~ dbern(mu.psi[k,j,i])

   # Create a loop to estimate detection for species i at stand j during sampling period k.
```

```
for (l in nvisit.start[j,k]:nvisit) {

logit(p[l,k,j,i]) <- b.0[i]  + b.1997[i]*year.1997[j] + b.1998[i]*year.1998[j] + b.trt[i]*trt[k]
+ b.date[i]*Julian.date.mat[l,k,j] + b.date.2[i]*Julian.date.mat[l,k,j]*Julian.date.mat[l,k,j]

mu.p[l,k,j,i] <- min(0.99, max(p[l,k,j,i], 0.01))*Z[k,j,i]
X[l,k,j,i] ~ dbern(mu.p[l,k,j,i])
                        }
                   }
              }
        }

    # Loop for all species in year 2-3
      for (i in 1:nspecies) {

            for (j in 2:nyear){


              # Create a loop to estimate the Z matrix (true occurrence for species i at stand j.
                    for (k in 1:nsite[j]) {



logit(psi[k,j,i]) <-  a.0[i] + rho[i]*Z[k,j-1,i] + a.1997[i]*year.1997[j] +
               a.1998[i]*year.1998[j] + a.trt[i]*trt[k]  + a.Winema[i]*district[k]+
               a.1997.trt[i]*year.1997[j]*trt[k] + a.1998.trt[i]*year.1998[j]*trt[k]
mu.psi[k,j,i] <- min(0.99, max(psi[k,j,i], 0.01))
Z[k,j,i] ~ dbern(mu.psi[k,j,i])

         # Create a loop to estimate detection for species i at stand j during sampling period k.
                        for (l in nvisit.start[j,k]:nvisit) {

logit(p[l,k,j,i]) <- b.0[i]  + b.1997[i]*year.1997[j] + b.1998[i]*year.1998[j] + b.trt[i]*trt[k]
+ b.date[i]*Julian.date.mat[l,k,j] +b.date.2[i]*Julian.date.mat[l,k,j]*Julian.date.mat[l,k,j]

mu.p[l,k,j,i] <- min(0.99, max(p[l,k,j,i], 0.01))*Z[k,j,i]
X[l,k,j,i] ~ dbern(mu.p[l,k,j,i])

                        }
                   }
              }
          }


        # Create a loop to determine point level richness estimates for the
        #  whole community.
            for (j in 1:nyear){
                    for (k in 1:nsite[j]) {
                    SiteRichness[k,j] <- sum(Z[k,j,])
                    }
      }


        # Calculation of species specific treatment effects
        for (i in 1:nspecies) {
             trt.1996[i] <-  a.trt[i]
             trt.1997[i] <-  a.trt[i]+ a.1997[i] + a.1997.trt[i]
             trt.1998[i] <-  a.trt[i]+ a.1998[i] + a.1998.trt[i]
      }



# Calculation of species richness separately by district and treatment

 for (j in 1:nyear){

SiteRichness.Winema.trt[j] <- inprod(SiteRichness[1:nsite[j],j],
                              Winema.trt[1:nsite[j]])/sum(Winema.trt[1:nsite[j]])

SiteRichness.Winema.control[j] <- inprod(SiteRichness[1:nsite[j],j],
                                  Winema.cont[1:nsite[j]])/sum(Winema.cont[1:nsite[j]])
```

```
SiteRichness.Fremont.trt[j] <- inprod(SiteRichness[1:nsite[j],j],
                              Fremont.trt[1:nsite[j]])/sum(Fremont.trt[1:nsite[j]])

SiteRichness.Fremont.control[j] <- inprod(SiteRichness[1:nsite[j],j],
                              Fremont.cont[1:nsite[j]])/sum(Fremont.cont[1:nsite[j]])
            }



for(j in 1:nyear){
    for (i in 1:nspecies){
            for(k in 1:22){
            Z.Fremont.cont[k,j,i] <-   Z[k,j,i]*Fremont.cont[k]
            Z.Fremont.trt[k,j,i] <-   Z[k,j,i]*Fremont.trt[k]
            Z.Winema.cont[k,j,i] <-   Z[k,j,i]*Winema.cont[k]
            Z.Winema.trt[k,j,i] <-   Z[k,j,i]*Winema.trt[k]
         }
    }
}

# Calculating turnover and extinction for each site
for(j in 2:nyear){
    for (i in 1:nspecies){
            for(k in 1:22){
            TO.num.Fremont.cont[k,j,i] <-   Z.Fremont.cont[k,j,i]*(1-Z.Fremont.cont[k,j-1,i])
            Ext.num.Fremont.cont[k,j,i] <-  (1-Z.Fremont.cont[k,j,i])*Z.Fremont.cont[k,j-1,i]
            Denom.Fremont.cont[k,j,i] <-   Z.Fremont.cont[k,j-1,i]

            TO.num.Fremont.trt[k,j,i] <-   Z.Fremont.trt[k,j,i]*(1-Z.Fremont.trt[k,j-1,i])
            Ext.num.Fremont.trt[k,j,i] <-  (1-Z.Fremont.trt[k,j,i])*Z.Fremont.trt[k,j-1,i]
            Denom.Fremont.trt[k,j,i] <-   Z.Fremont.trt[k,j-1,i]


            TO.num.Winema.cont[k,j,i] <-   Z.Winema.cont[k,j,i]*(1-Z.Winema.cont[k,j-1,i])
            Ext.num.Winema.cont[k,j,i] <-  (1-Z.Winema.cont[k,j,i])*Z.Winema.cont[k,j-1,i]
            Denom.Winema.cont[k,j,i] <-   Z.Winema.cont[k,j-1,i]

            TO.num.Winema.trt[k,j,i] <-   Z.Winema.trt[k,j,i]*(1-Z.Winema.trt[k,j-1,i])
            Ext.num.Winema.trt[k,j,i] <-  (1-Z.Winema.trt[k,j,i])*Z.Winema.trt[k,j-1,i]
            Denom.Winema.trt[k,j,i] <-   Z.Winema.trt[k,j-1,i]

         }
        TO.num2.Fremont.cont[i,j] <-sum(TO.num.Fremont.cont[,j,i])
        Ext.num2.Fremont.cont[i,j] <-sum(Ext.num.Fremont.cont[,j,i])
        Denom2.Fremont.cont[i,j]  <-sum(Denom.Fremont.cont[,j,i])

        TO.num2.Fremont.trt[i,j] <-sum(TO.num.Fremont.trt[,j,i])
        Ext.num2.Fremont.trt[i,j] <-sum(Ext.num.Fremont.trt[,j,i])
        Denom2.Fremont.trt[i,j]  <-sum(Denom.Fremont.trt[,j,i])


        TO.num2.Winema.cont[i,j] <-sum(TO.num.Winema.cont[,j,i])
        Ext.num2.Winema.cont[i,j] <-sum(Ext.num.Winema.cont[,j,i])
        Denom2.Winema.cont[i,j]  <-sum(Denom.Winema.cont[,j,i])

        TO.num2.Winema.trt[i,j] <-sum(TO.num.Winema.trt[,j,i])
        Ext.num2.Winema.trt[i,j] <-sum(Ext.num.Winema.trt[,j,i])
        Denom2.Winema.trt[i,j]  <-sum(Denom.Winema.trt[,j,i])


    }
  TO.Fremont.cont[j] <-sum(TO.num2.Fremont.cont[,j])/sum(Denom2.Fremont.cont[,j])
  Ext.Fremont.cont[j] <-sum(Ext.num2.Fremont.cont[,j])/sum(Denom2.Fremont.cont[,j])

  TO.Fremont.trt[j] <-sum(TO.num2.Fremont.trt[,j])/sum(Denom2.Fremont.trt[,j])
  Ext.Fremont.trt[j] <-sum(Ext.num2.Fremont.trt[,j])/sum(Denom2.Fremont.trt[,j])

  TO.Winema.cont[j] <-sum(TO.num2.Winema.cont[,j])/sum(Denom2.Winema.cont[,j])
  Ext.Winema.cont[j] <-sum(Ext.num2.Winema.cont[,j])/sum(Denom2.Winema.cont[,j])
```

```
  TO.Winema.trt[j] <-sum(TO.num2.Winema.trt[,j])/sum(Denom2.Winema.trt[,j])
  Ext.Winema.trt[j] <-sum(Ext.num2.Winema.trt[,j])/sum(Denom2.Winema.trt[,j])

}



# dissimalarity index to compare the species composition within each year and district between
treatment and control
# calculating the occupancy for each species x year for each district x treatment
for(j in 1:nyear){
    for (i in 1:nspecies){
            Z.Fremont.cont.stand[j,i] <-  step(sum(Z.Fremont.cont[,j,i])-1)
            Z.Fremont.trt.stand[j,i] <-  step(sum(Z.Fremont.trt[,j,i])-1)
            Z.Winema.cont.stand[j,i] <-  step(sum(Z.Winema.cont[,j,i])-1)
            Z.Winema.trt.stand[j,i] <-  step(sum(Z.Winema.trt[,j,i])-1)

            Z.Fremont[j,i] <- Z.Fremont.cont.stand[j,i]*Z.Fremont.trt.stand[j,i]
            Z.Winema[j,i] <- Z.Winema.cont.stand[j,i]*Z.Winema.trt.stand[j,i]
            }

    Similarity.Fremont[j] <- 2*sum(Z.Fremont[j,])/(sum(Z.Fremont.cont.stand[j,]) +
                             (sum(Z.Fremont.trt.stand[j,])))
    Similarity.Winema[j] <- 2*sum(Z.Winema[j,])/(sum(Z.Winema.cont.stand[j,]) +
                             (sum(Z.Winema.trt.stand[j,])))

    }


# all pairwise sites dissimalarity index to compare the species composition within each year and
district between treatment and control

for (j in 1:nyear){
      for (Fre.cont in 1:6){
          for (Fre.trt in 7:12){

            for (i in 1:nspecies){

        Pairwise.Similarity.Fre.temp[j, i, Fre.cont, Fre.trt] <- Z[Fre.cont,j, i]*Z[Fre.trt,j, i]
            }

        Pairwise.Similarity.Fre[j, Fre.cont, Fre.trt] <- 2*sum(Pairwise.Similarity.Fre.temp[j, ,
                              Fre.cont, Fre.trt])/(sum( Z[Fre.cont,j, ]) + sum(Z[Fre.trt,j,]))
        }
    }
}

for (j in 1:nyear){
      for (Win.cont in 13:18){
          for (Win.trt in 19:22){

            for (i in 1:nspecies){

        Pairwise.Similarity.Win.temp[j, i, Win.cont, Win.trt] <- Z[Win.cont,j, i]*Z[Win.trt,j, i]
            }

        Pairwise.Similarity.Win[j, Win.cont, Win.trt] <- 2*sum(Pairwise.Similarity.Win.temp[j, ,
                              Win.cont, Win.trt])/(sum( Z[Win.cont,j, ]) + sum(Z[Win.trt,j,]))
        }
    }
}

for (j in 1:nyear){
      for (Fre.cont.1 in 1:6){
           for (Fre.cont.2 in 1:6){

            for (i in 1:nspecies){

        Pairwise.Similarity.Fre.cont.temp[j, i, Fre.cont.1, Fre.cont.2] <- Z[Fre.cont.1,j, i]*
               Z[Fre.cont.2,j, i]
            }
```

```
        Pairwise.Similarity.Fre.cont[j, Fre.cont.1, Fre.cont.2] <-
                2*sum(Pairwise.Similarity.Fre.cont.temp[j, , Fre.cont.1, Fre.cont.2])/(sum(
                Z[Fre.cont.1,j, ]) + sum(Z[Fre.cont.2,j,]))
        }
    }
}

for (j in 1:nyear){
for (Fre.trt.1 in 7:12){
           for (Fre.trt.2 in 7:12){

            for (i in 1:nspecies){

            Pairwise.Similarity.Fre.trt.temp[j, i, Fre.trt.1, Fre.trt.2] <- Z[Fre.trt.1,j, i]*
                                            Z[Fre.trt.2,j, i]
            }

        Pairwise.Similarity.Fre.trt[j, Fre.trt.1, Fre.trt.2] <-
                2*sum(Pairwise.Similarity.Fre.trt.temp[j, , Fre.trt.1, Fre.trt.2])/(sum(
                Z[Fre.trt.1,j, ]) + sum(Z[Fre.trt.2,j,]))
        }
    }
}

for (j in 1:nyear){
      for (Win.cont.1 in 13:18){
           for (Win.cont.2 in 13:18){

            for (i in 1:nspecies){

         Pairwise.Similarity.Win.cont.temp[j, i, Win.cont.1, Win.cont.2] <- Z[Win.cont.1,j, i]*
                Z[Win.cont.2,j, i]
            }

         Pairwise.Similarity.Win.cont[j, Win.cont.1, Win.cont.2] <-
                        2*sum(Pairwise.Similarity.Win.cont.temp[j, , Win.cont.1,
                        Win.cont.2])/(sum( Z[Win.cont.1,j, ]) + sum(Z[Win.cont.2,j,]))
        }
    }
}

for (j in 1:nyear){
      for (Win.trt.1 in 19:22){
           for (Win.trt.2 in 19:22){

            for (i in 1:nspecies){

            Pairwise.Similarity.Win.trt.temp[j, i, Win.trt.1, Win.trt.2] <- Z[Win.trt.1,j, i]*
                        Z[Win.trt.2,j, i]
            }

        Pairwise.Similarity.Win.trt[j, Win.trt.1, Win.trt.2] <-
                        2*sum(Pairwise.Similarity.Win.trt.temp[j, , Win.trt.1, Win.trt.2])/(sum(
                        Z[Win.trt.1,j, ]) + sum(Z[Win.trt.2,j,]))
        }
    }
}


}
```

```
# ---------------------------------------------------------------------------
# fit the WinBUGS model
# ---------------------------------------------------------------------------


# Specifying parameters to track in WinBUGS

  params <- c('mu.a.0', 'mu.rho', 'mu.a.1997', 'mu.a.1998', 'mu.a.trt', 'mu.a.Winema',
       'mu.a.1997.trt', 'mu.a.1998.trt', 'mu.b.0', 'detection.prob.int', 'mu.b.1997',
       'mu.b.1998', 'mu.b.trt', 'mu.b.date', 'mu.b.date.2',  'pi0',

       'sig2.a.0','sig2.rho', 'sig2.a.1997', 'sig2.a.1998', 'sig2.a.trt', 'sig2.a.Winema',
       'sig2.a.1997.trt',  'sig2.a.1998.trt','sig2.b.0', 'sig2.b.1997', 'sig2.b.1998',
       'sig2.b.trt', 'sig2.b.date', 'sig2.b.date.2',

       'a.0', 'rho', 'a.1997', 'a.1998', 'a.trt', 'a.Winema', 'a.1997.trt',  'a.1998.trt',
       'b.0', 'b.1997', 'b.1998', 'b.trt', 'b.date', 'b.date.2', 'SiteRichness',
                      'trt.1996', 'trt.1997', 'trt.1998',

       'SiteRichness.Winema.trt', 'SiteRichness.Winema.control', 'SiteRichness.Fremont.trt',
       'SiteRichness.Fremont.control','Similarity.Fremont', 'Similarity.Winema',


       'Pairwise.Similarity.Fre.cont', 'Pairwise.Similarity.Fre.trt',
       'Pairwise.Similarity.Win.cont', 'Pairwise.Similarity.Win.trt',
       'Pairwise.Similarity.Fre', 'Pairwise.Similarity.Win',

        'TO.Fremont.cont', 'Ext.Fremont.cont', 'TO.Fremont.trt', 'Ext.Fremont.trt',
        'TO.Winema.cont', 'Ext.Winema.cont', 'TO.Winema.trt', 'Ext.Winema.trt')



# Writing model file

write.model(chemult.occ.model, "D:\\Giovanini data\\BUGS Models\\chemult.occ.model.txt")
model.file <- "D:\\Giovanini data\\BUGS Models\\chemult.occ.model.txt"






# Specify the initial values

inits= function(){list(mu.a.0=rnorm(1, 0, .1), mu.rho=rnorm(1, 0, .1), mu.a.1997=rnorm(1, 0, .1),
      mu.a.1998=rnorm(1, 0, .1), mu.a.trt=rnorm(1, 0, .1), mu.a.Winema=rnorm(1, 0, .1),
      mu.a.1997.trt=rnorm(1, 0, .1), mu.a.1998.trt=rnorm(1, 0, .1),  detection.prob.int =
      rbeta(1, 0.90, 1.85), mu.b.1997=rnorm(1, 0, .1), mu.b.1998=rnorm(1, 0, .1),
      mu.b.trt=rnorm(1, 0, .1), mu.b.date=rnorm(1, 0, .1), mu.b.date.2=rnorm(1, 0, .1),
      pi0=runif(1,0,1),

      sig2.a.0=rgamma(1, .5, .5), sig2.rho=rgamma(1, .5, .5), sig2.a.1997=rgamma(1, .5, .5),
      sig2.a.1998=rgamma(1,.5,.5), sig2.a.trt=rgamma(1, .5,.5), sig2.a.Winema=rgamma(1,.5,.5),
      sig2.a.1997.trt=rgamma(1, .5, .5),  sig2.a.1998.trt=rgamma(1, .5, .5),
      sig2.b.0=rgamma(1, .5, .5), sig2.b.1997=rgamma(1, .5, .5), sig2.b.1998=rgamma(1, .5, .5),
      sig2.b.trt=rgamma(1, .5, .5), sig2.b.date=rgamma(1, .5, .5),
      sig2.b.date.2=rgamma(1, .5, .5),


      a.0=rnorm(nspecies, 0, .1),rho=rnorm(nspecies, 0, .1), a.1997=rnorm(nspecies, 0, .1),
      a.1998=rnorm(nspecies, 0,.1), a.trt=rnorm(nspecies, 0,.1), a.Winema=rnorm(nspecies,0,.1),
      a.1997.trt=rnorm(nspecies, 0, .1), a.1998.trt=rnorm(nspecies, 0, .1),
      b.0=rnorm(nspecies, 0, .1), b.1997=rnorm(nspecies, 0, .1), b.1998=rnorm(nspecies, 0, .1),
      b.trt=rnorm(nspecies,0,.1), b.date=rnorm(nspecies,0, .1), b.date.2=rnorm(nspecies, 0, .1),

      Z = array(rbinom(nyear*nspecies*24, size=1, prob=0.5), dim=c(24, nyear,nspecies)))
    }
```

```
# Packaging the data

data.chemult <- list(nyear=nyear, nsite=nsite, nspecies=nspecies, nvisit=nvisit,
               nvisit.start=nvisit.start, trt=trt, district=district, year.1997=year.1997,
               year.1998=year.1998, X=X,  Winema.trt=Winema.trt, Winema.cont=Winema.cont ,
               Fremont.trt=Fremont.trt, Fremont.cont=Fremont.cont,
               Julian.date.mat=Julian.date.mat)




# ---------------------------------------------------------------------------
# fit the WinBUGS model using the bugs function
# ---------------------------------------------------------------------------

chem <- bugs(data.chemult, inits, params, model.file, bugs.directory="D:/Program
               Files/WinBUGS14/",working.directory=NULL, clearWD=FALSE, over.relax=T,
                 debug=F, n.chains=3, n.iter=500000, n.burnin=250000, n.thin=50)


# Saving the results

dchem <- as.data.frame(chem$summary)
```
