# Supplementary material for: Effects of Management Intervention on Post-Disturbance Community Composition: An Experimental Analysis Using Bayesian Hierarchical Models
Source: PLoS One. 2013 Mar 22;8(3):e59900. doi: 10.1371/journal.pone.0059900 (PMC3606292; doi:10.1371/journal.pone.0059900)

Text S6: Posterior predictive checks to assess goodness of fit for Bayesian models.

To assess consistency between data and our models, we used posterior predictive checks (62). If the model provides adequate fit, then observed data should look like data simulated using the posterior predictive distribution. For our posterior predictive checks, we simulated a data set for each of our MCMC posterior samples of true occupancy and observed occupancy. For each treatment and year, we calculated the proportion of visits where each species was observed. This distribution is then compared to the same quantity from the actual dataset. A plot is shown, with CHSP as an example species, below. These checks did not reveal a lack of fit for our models. Histograms by treatment and year of the proportion of visits where CHSP was observed based on simulated datasets from our model fit. The red line shows proportion of visits where CHSP was observed for the actual dataset.

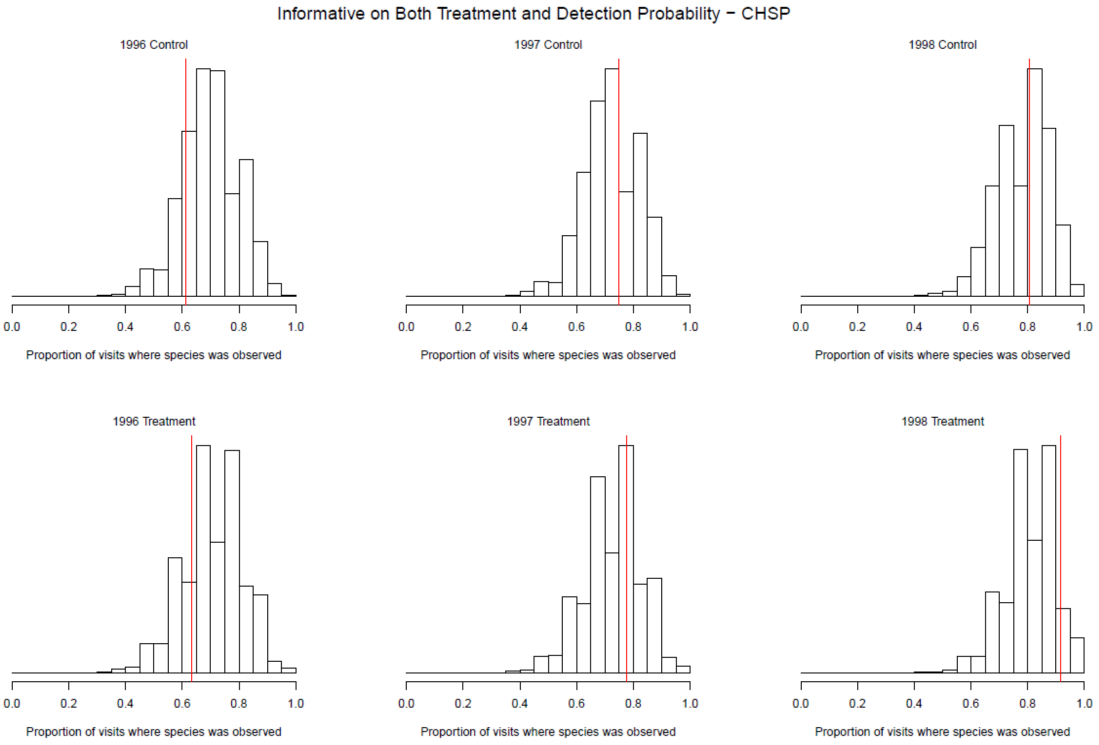

Supplement: Text S2 — Posterior predictive checks to assess goodness of fit for Bayesian models, Fremont and Winema National Forests, south-central Oregon, USA, 1996–1998. (PDF) [file pone.0059900.s006.pdf]
